# Global trends in research of glutamate in epilepsy during past two decades: A bibliometric analysis

**DOI:** 10.3389/fnins.2022.1042642

**Published:** 2022-10-20

**Authors:** Wei Wang, Runshi Gao, Zhiwei Ren, Dongju Yang, Ke Sun, Xiaoling Li, Suying Yan

**Affiliations:** ^1^Department of Pharmacy, Xuanwu Hospital, Capital Medical University, Beijing, China; ^2^Department of Functional Neurology, Xuanwu Hospital, Capital Medical University, Beijing, China; ^3^Xuanwu Hospital, Beijing Institute of Functional Neurosurgery, Capital Medical University, Beijing, China; ^4^Department of Neurology, Xuanwu Hospital, Capital Medical University, Beijing, China; ^5^Department of Functional Neurology, National Center for Children’s Health of China, Beijing Children’s Hospital, Capital Medical University, Beijing, China

**Keywords:** epilepsy, glutamate, bibliometric research, VOSviewer, CiteSpace, trends

## Abstract

Epilepsy affects more than 70 million people in the world. It is characterized by recurrent spontaneous seizures, and it is related to many neurological, cognitive, and psychosocial consequences. Glutamate neurotransmitter dysfunction has essential functions in the pathophysiology of epilepsy. In this work, bibliometric analysis was conducted to explore the trends, frontiers, and hotspots of the global scientific output of glutamate in epilepsy research in the past 20 years. The Science Citation Index Expanded of the Web of Science Core Collection (WoSCC) was searched to obtain information on publications and records published between 2002 and 2021. VOSviewer and CiteSpace were used to conduct bibliometric and visual analyses on the overall distribution of annual output, major countries, active institutions, journals, authors, commonly cited literature, and keywords. The impact and quality of the papers were assessed using the global citation score (GCS). Four thousand eight hundred ninety-one publications were retrieved in total. During the past two decades, the number of publications (Np) associated with glutamate in epilepsy has risen yearly. The United States has published the most papers; its H-index and number of citations are also the highest. The League of European Research Universities (LERU) was the most productive institution. In 2016, the total score of the paper written by Zhang Y was 854, ranking first. The keywords that appear most frequently are “epilepsy,” “glutamate,” “temporal lobe epilepsy (TLE),” “hippocampus,” and “seizures.” This study showed that although the publications related to epileptic glutamate fluctuated slightly, the Np increased overall. The United States is a great creator and influential country in this field. The first three authors are Eid, T., Aronica, E., and Smolders, I. “spectrum,” “animal model,” “inflammation,” “mutation,” “dysfunction,” and “prefrontal cortex” are increasing research hotspots. By recognizing the most critical indicators (researchers, countries, research institutes, and journals of glutamate release in epilepsy research), the research hotspot of glutamate in epilepsy could help countries, scholars, and policymakers in this field enhance their understanding of the role of glutamate in epilepsy and make decisions.

## Introduction

More than 70 million people suffer from epilepsy all over the world, the features of which are recurrent spontaneous seizures and are accompanied by many neurological, cognitive, and psychosocial consequences. Although antiepileptic drugs (AEDs) are first-line therapeutic drugs, they are ineffective for about one-third of patients with drug-resistant epilepsy (DRE) (Czornyj et al., 2022). Patients with DRE usually experience decreased quality of life, underemployment, and increased mortality. Glutamate has been shown to be neurotoxic in excess, causing neuronal death and a variety of neuropsychiatric disorders, including epilepsy (Green et al., 2021). Glutamate mediates most excitatory neurotransmission in the mammalian CNS by activating ionotropic glutamate receptors [N-methyl-D-aspartic acid (NMDA) and α-amino-3-hydroxy-5-methyl-4-isoxazole-propionicacid (AMPA)/kainate receptors] and metabotropic glutamate receptors (mGluRs) (Hansen et al., 2021). Mutations in the NMDA receptor (NMDAR) and AMPA receptor (AMPAR) genes may cause epilepsy in humans (Lemke et al., 2013, 2016; Lesca et al., 2013; Piard et al., 2018). Activating mGluRs represents a potential mechanism for regulating glutamatergic signaling in epilepsy (Yu et al., 2019; Gregory and Goudet, 2021; Celli et al., 2022; Kovalenko et al., 2022). Therefore, quantitatively analyzing the current situation, focus areas, and prospects of glutamate in epilepsy are very important. Bibliometrics, based on mathematical and statistical methods, can quantify and comprehensively analyze the publications (Dong et al., 2022). In addition, bibliometrics is a common and mature method to investigate the research status of the discipline (Chen et al., 2021). Based on the evaluation of database and literature characteristics, bibliometrics could estimate the development trend in scientific documents and expose the research frontier as a convenient technology. In addition, it could provide reliable data, which could be used as a reference for experimental strategies and financing decisions (Cheng et al., 2022b). Furthermore, based on bibliometrics, the hotspots of research can be evaluated and predicted (Deng et al., 2022). There were bibliometric fruits in metformin (Song et al., 2021), cancer photodynamic therapy (Cheng et al., 2022a), macrophages associated with acute lung injury (Wang S. et al., 2021) and et al. However, no bibliometric study has been conducted on glutamate in epilepsy. Therefore, the present study aimed to work on an in-depth discussion on glutamate research in epilepsy to evaluate the research status and hotspots in this field and our work is expected to encourage more important research and benefit scholars in terms of shaping research directions in the future.

## Materials and methods

### Retrieval strategies and data sources

**FIGURE 1:**
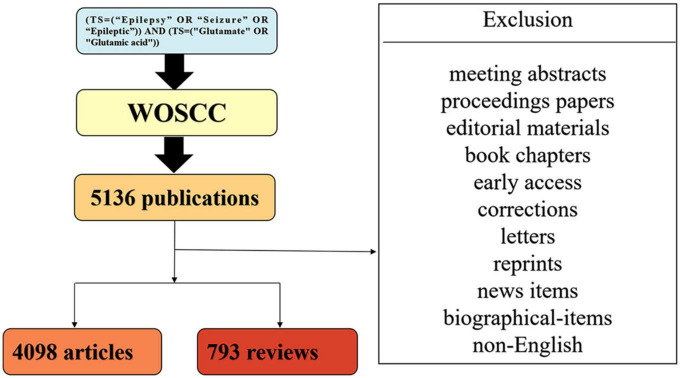
Flowchart of the filtering process.

Bibliometric analysis was conducted with the Science Citation Index Expanded (SCI-expanded) of the Web of Science Core Collection (WoSCC). A literature search was conducted on May 29, 2022, to avoid deviations because database renewal is rapid. The timeline was set to 2002–2021. The retrieval terms were as follows: [TS = (“Epilepsy” OR “Seizure” OR “Epileptic”)] AND [TS = (“Glutamate” OR “Glutamic acid”)]. Only original papers in English were involved. Therefore, 4,891 papers were analyzed in our study. Figure 1 shows the detailed filtering.

### Data download

First, raw data extraction was completed through the SCI Extended database. The information included the Np and citations, references, country/region, affiliate, author, H-index, year of publication, journal, and keywords. Finally, we imported data into VOSviewer and CiteSpace for further analysis.

### Bibliometric analysis

The fitting polynomial model was used to predict the annual Np and further explain the yearly literature amount change. The variable f (x) represents the number of studies per year and X represents the year of publication. Besides, a network was constructed through the VOSviewer software 1.6.10 to obtain more comprehensive result information based on co-citation and co-occurrence (van Eck and Waltman, 2010; Baier-Fuentes et al., 2020). When the third entry references two entries simultaneously, a co-reference is defined. The co-occurrence of keywords measures the most frequently occurring keywords in the same literature (Merigo et al., 2018). The analysis of co-cited references and co-occurrence keywords explains the research hotspots related to glutamate in epilepsy. Cluster analysis, timeline, references, and keyword citation bursts are the tools utilized by CiteSpace 6.1.R2 in the visual examination of the knowledge domain and emerging trends (Luo et al., 2021). References, keywords, and identified crucial research areas for glutamate in epilepsy are categorized by cluster analysis. In identifying emerging research trends, bursts of keywords and references are regularly used.

## Results

### Summary of papers on glutamate in epilepsy

This study retrieved four thousand eight hundred ninety-one publications through the retrieval strategy. The Nc of all publications was 158,830, and the average Nc of each article was 36.58. The H-index for all papers was 163.

### The trend of published papers each year

**FIGURE 2:**
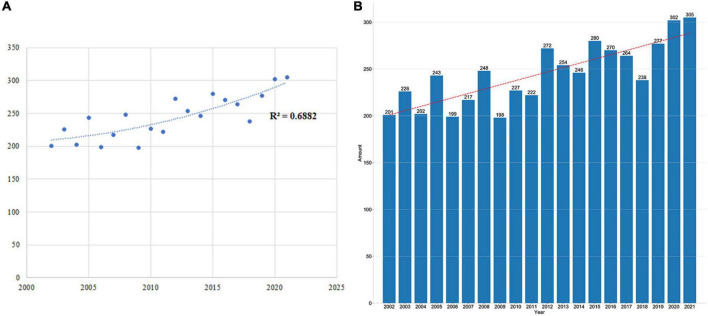
**(A)** Curve fitting between Np each year and publication year (*R*^2^ = 0.6882). **(B)** Np in each year over the past 20 years.

A fitting curve was shown in Figure 2A to clarify the trend of paper publishing volume each year. No remarkable correlation was found between the publication year and annual Np; the correlation coefficient R^2^ was 0.6882, as shown in Figure 2A. Figure 2B presents the Np of each year associated with glutamate in epilepsy. Generally, despite 20-year fluctuations, the number of papers each year raised from 201 in 2002 to 305 in 2021. These findings indicated that research on glutamate in epilepsy has entered a phase of rapid development and has become the center of attention.

### Performance of countries/regions

The ten countries/regions with the most publications were ranked based on Np (Table 1). The USA showed the most significant number of articles (1,680), followed by China (509) and Germany (468). The total Nc of the USA was 79,929, followed by Germany (21,099) and Italy (16,183). Besides, the USA ranked first on the H-index (132), which was twice the figure for England (66). Although England had moderately lower Np than Japan, it had higher Nc and H-index.

**TABLE 1:** Top 10 countries/regions with most papers.

Rank	Country/Region	Np	% of (4,891)	Nc	H-index	Total link strength
1	USA	1,680	34.35%	79,929	132	918
2	China	509	10.41%	10,856	46	174
3	Germany	468	9.57%	21,088	70	486
4	Italy	375	7.67%	16,183	67	359
5	Japan	359	7.34%	11,617	55	122
6	England	320	6.54%	16,035	66	440
7	Canada	219	4.48%	10,507	51	217
8	France	216	4.42%	9,785	51	272
9	Brazil	168	3.43%	3,651	31	74
10	Spain	149	3.05%	6,007	43	144

**FIGURE 3:**
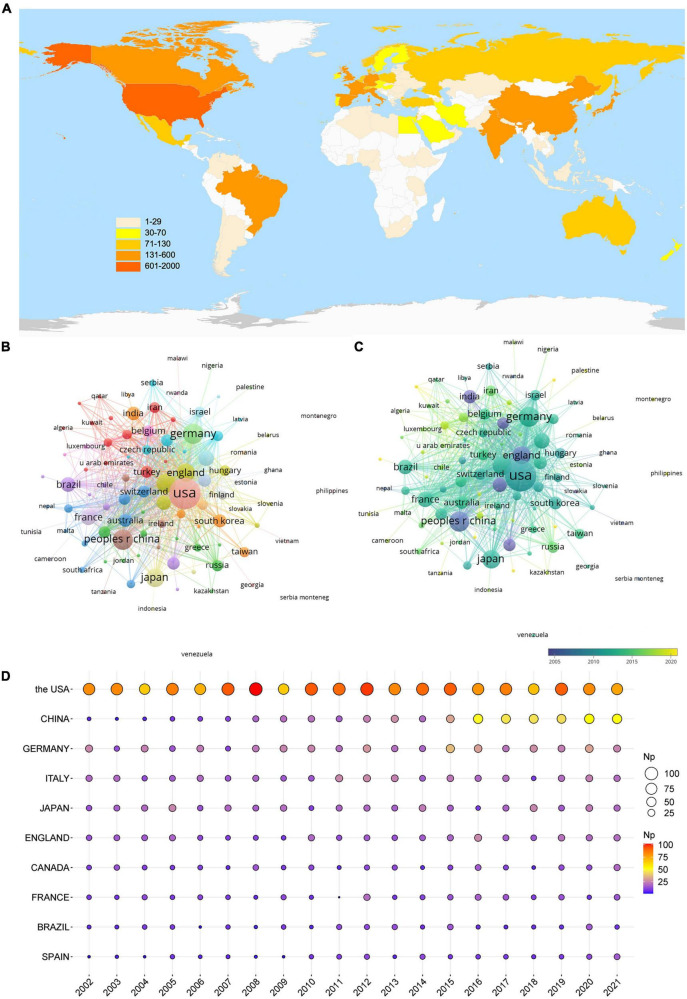
Leading countries in glutamate in epilepsy. **(A)** Global geographical distribution of the publication. **(B)** Co-occurrence of countries. **(C)** Visualization of countries based on APY. **(D)** The annual publication of the top 10 countries.

The international cooperation network demonstrated how closely the various nations cooperate (Figure 3B). The USA, Germany, England, and Italy showed high total link strength, indicating their close cooperation with other countries. Countries represented by blue nodes, such as India, Spain, England, and China, posted publications earlier than the countries represented by green nodes, such as the USA, Japan, Italy, and Germany (Figure 3C). The countries represented by yellow nodes, such as Saudi Arabia and Malaysia, started their research later, so they published fewer articles and had smaller nodes. Figure 3D shows that over the past 20 years, the USA has had a high annual Np, China has had a progressively higher annual Np, and other countries have fluctuated in their annual Np.

### Affiliation performance

**FIGURE 4:**
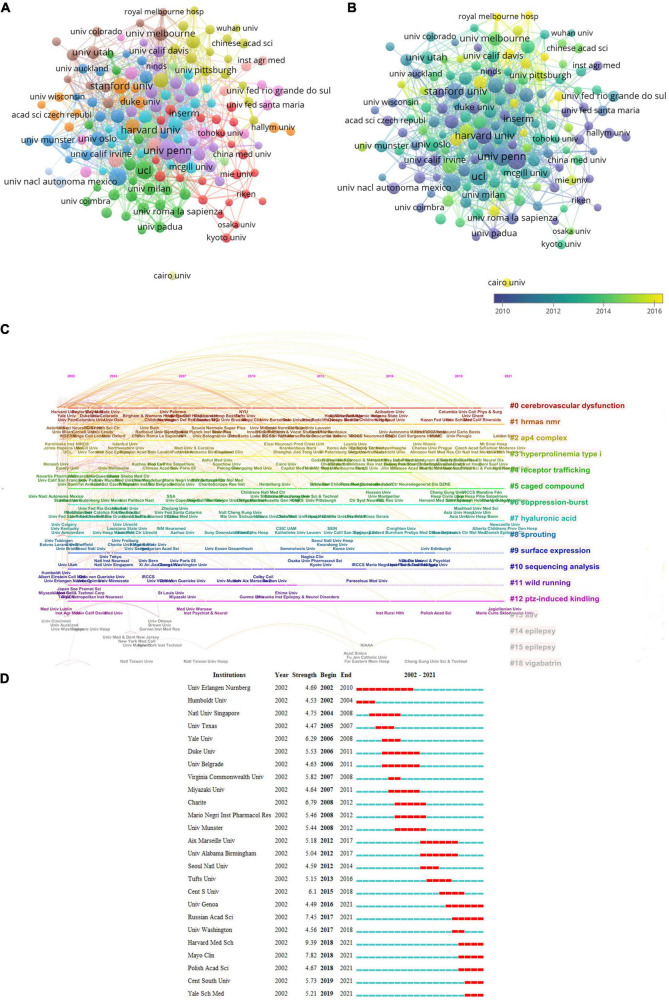
Visualization of institutes. **(A)** Co-occurrence of institutes. **(B)** Visualization of countries based on APY. **(C)** Timeline distribution of the top 19 clusters. **(D)** Top 25 institutes with strongest burstness.

The top 10 institutes with the most Np associated with glutamate for epilepsy are shown in Table 2. The League of European Research Universities (LERU) possessed the most publications (384); the next was the University of California System (188) and UDICE French Research Universities (156). LERU also ranked first for the Nc (19,803) and the H-index. Institutional cooperation was relatively close (Figure 4A). The University of Pennsylvania had the most collaborative efforts with other institutions, followed by Yale University, Harvard University, and University College London. These institutions, which are at the forefront of collaboration in research into the role of glutamate in epilepsy, release publications earlier (Figure 4B). The study represented by the clusters “high-resolution magic-angle spinning nuclear magnetic resonance,” “adaptor protein complex-4,” “hyperprolinemia type I,” “caged compound,” and “hyaluronic acid” remains influential and continues to have a research buzz (Figure 4C). Among the 25 most referenced institutes that have remained viable thus far are the University of Genoa, Russian Academy of Sciences, Harvard Medical School, Mayo Clinic, Polish Academy of Sciences, Central South University, and Yale School of Medicine (Figure 4D).

**TABLE 2:** Top 10 affiliations with most publications.

Rank	Affiliations	Country	Np	Nc	H-index
1	League of European Research Universities (LERU)	Germany	384	19,803	74
2	University of California System	USA	188	12,857	58
3	UDICE French Research Universities	France	156	7,706	48
4	Institut National De La Sante Et De La Recherche Medicale (INSERM)	France	153	6,387	44
5	University of London	England	143	8,982	48
6	University College London	England	116	7,057	43
7	Harvard University	USA	96	5,428	41
8	Centre National De La Recherche Scientifique (CNRS)	France	89	3,990	32
9	Yale University	USA	85	4,202	38
10	National Institutes of Health (NIH)	USA	82	4,804	39

### Journal performance

Epilepsia (227 publications, IF: 6.74) published the most articles about glutamate in epilepsy, followed by Epilepsy Research (157 publications, IF: 2.991) and Brain Research (149 publications, IF: 3.61). Except for Epilepsy Behavior (IF: 2.991), and rest of the top 10 journals had high IF (IF > 3.000). Besides, the Journal of Neuroscience (IF = 6.709) ranked first for H-index and Nc (Table 3).

**TABLE 3:** Top 10 journals.

Rank	Journal	Np	Nc	H-index	IF (2021)
1	Epilepsia	227	8,645	55	6.74
2	Epilepsy research	157	4,227	32	2.991
3	Brain research	149	3,243	32	3.61
4	Journal of neuroscience	120	9,437	58	6.709
5	Neuroscience	120	4,200	37	3.708
6	Neuropharmacology	115	4,115	33	5.273
7	Epilepsy behavior	91	2,187	26	3.337
8	Journal of neurochemistry	81	3,524	35	5.546
9	Neurobiology of disease	81	3,318	34	7.046
10	Neurochemical research	81	2,078	25	4.414

### Author performance

**FIGURE 5:**
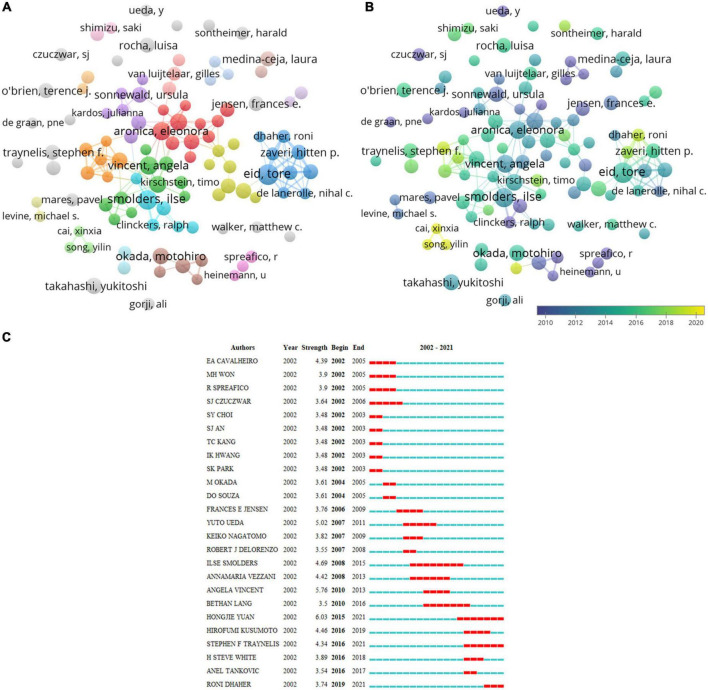
Visualization of author analysis. **(A)** Co-occurrence of authors. **(B)** Visualization of countries based on APY. **(C)** Top 25 authors with the most vigorous citation bursts.

Table 4 lists the top 10 productive authors. Eid, T. (33) from Yale University was in the first place in the investigation of glutamate in epilepsy, and the next was Aronica, E. (32) from the University of Amsterdam in the Netherlands and Smolders, I. (30) from Vrije Universiteit Brussel in Belgium. Vincent, A and Vezzani, A had high Nc (3,228 and 3,183, respectively). Their work has attracted more scholars’ attention. The degree of cooperation among authors is shown in Figure 5A. Eid, T., Aronica, E., Smolders, I., and Vincent, A. all occupied central positions in their respective groups. They started research into the role of glutamate in epilepsy in 2012, whereas Dhaher, R., Cai, XX., and Song, YL. began relevant research after 2018 (Figure 5B). Among the 25 most referenced authors that have remained viable are Yuan, HJ., Traynelis, S., and Dhaher, R. (Figure 5C).

**TABLE 4:** Top 10 authors in terms of Np.

Rank	Author	Affiliations	Country	Np	Nc	H-index
1	Eid T	Yale University	USA	33	1,312	19
2	Aronica E	University of Amsterdam	Netherlands	32	2,424	23
3	Smolders I	Vrije Universiteit Brussel	Belgium	30	959	18
4	Vincent A	University of Oxford	England	29	3,228	22
5	Vezzani A	IRCCS Mario Negri	Italy	25	3,183	23
6	Elger CE	University of Bonn	Germany	25	1,350	17
7	Steinhauser C	University of Bonn	Germany	23	1,929	17
8	Sonnewald U	Norwegian University of Science and Technology (NTNU)	Norway	23	718	16
9	Kang TC	Hallym University	South Korea	23	639	14
10	Ueda Y	University of Miyazaki	Japan	22	452	11

### Analysis of highly cited papers

Table 5 presents the total citations of articles in descending order. Most of the top 10 highly cited papers were published between 2012 and 2019. The writing in Neuron ranked first (Zhang et al., 2016). This paper developed a method in which astrocytes from healthy people and patients were purified (e.g., epilepsy and glioblastoma) and cultured in serum-free conditions (Zhang et al., 2016). It was followed by the European Journal of Pharmacology (Mehta et al., 2013) and PNAS (Pascual et al., 2012).

**TABLE 5:** Top 10 highest cited articles.

Rank	References	Article	IF (2021)	Total citation	Type of study
1	Zhang et al., 2016	Purification and characterization of progenitoer and mature human astrocytes reveals transcriptional and functional differences with mouse. Neuron 2016 Jan 06;89(1):37–53	18.688	854	Article
2	Mehta et al., 2013	Excitotoxicity: Bridge to various triggers in neurodegenerative disorders. European journal of pharmacology 2013 Jan 05;698(1–3):6–18	5.195	393	Review
3	Pascual et al., 2012	Microglia activation triggers astrocyte-mediated modulation of excitatory neurotransmission. Proceedings of the National Academy of Sciences of the United States of America 2012 Jan 24;109(4):E197–205	12.779	388	Article
4	Vezzani et al., 2013	Epilepsy and brain inflammation. Experimental neurology 2013 Jun;244:11–21	5.62	333	Review
5	Vezzani and Viviani, 2015	Neuromodulatory properties of inflammatory cytokines and their impact on neuronal excitability. Neuropharmacology 2015 Sep;96(Pt A):70–82	5.273	290	Review
6	Xanthos and Sandkuehler, 2014	Neurogenic neuroinflammation: Inflammatory CNS reactions in response to neuronal activity. Nature reviews. Neuroscience. 2014 Jan; 15 (1):43–53	38.755	297	Review
7	Parsons and Raymond, 2014	Extrasynaptic NMDA receptor involvement in central nervous system disorders. Neuron. 2014 Apr 16; 82 (2):279–93	18.688	285	Review
8	Olson et al., 2018	The Gut microbiota mediates the anti-seizure effects of the ketogenic diet. Cell. 2018 06 14; 173 (7):1728–1741.e13.	66.85	226	Article
9	Deepmala et al., 2015	Clinical trials of N-acetylcysteine in psychiatry and neurology: A systematic review. Neuroscience and biobehavioral reviews 2015 Aug;55:294–321	9.052	234	Review
10	Venkataramani et al., 2019	Glutamatergic synaptic input to glioma cells drives brain tumor progression. Nature. 2019 09; 573 (7775):532–538	69.504	204	Article

### Global citation score analysis

**FIGURE 6:**
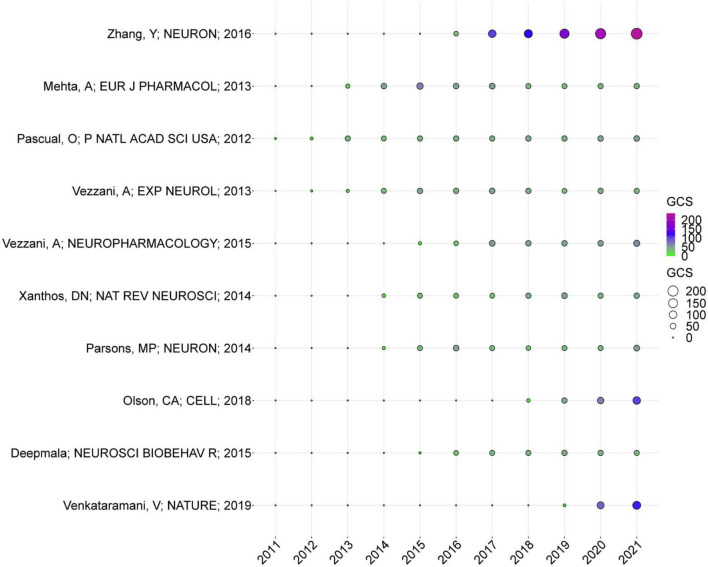
Annual global citation score (GCS) of the top 10 publications.

Figure 6 depicts the annual global status of publications which has high GCSs. GCS of articles written by Zhang et al. (2016) was 236, which was the first. Based on their research, unlike previously observed mouse astrocytes, mature human astrocytes showed strong calcium responses to glutamate stimulation via mGluRs. These findings indicate that adult human astrocytes have evolved to detect synaptic activity and potential responses more effectively (Zhang et al., 2016). Recently, Venkataramani et al. (2019) received more GCS for their research, which examined the mechanism behind the notion that excessive neuronal activity during seizures may stimulate the progression of brain tumors. As a result of glutamatergic synaptic input to glioma cells, brain tumor progression is facilitated by their effect on calcium communication within the network of tumor cells linked by microtubules of glioma cells (Venkataramani et al., 2019). Additionally, the works of Olson et al. (2018), which demonstrated that the ketogenic diet has a favorable effect on seizures by regulating the γ-aminobutyric acid (GABA)/glutamate ratio in the hippocampus through the gut microbiota, had increased GCS in recent years. Work of Vezzani et al. (2013), Xanthos and Sandkuehler (2014), and Vezzani and Viviani (2015), which summarized the function of specific soluble inflammatory mediators in the pathogenesis of epilepsy and the fundamental molecular mechanisms in glia-neuron interactions, shed light on how brain inflammation leads to neuronal hyper-excitability in epilepsy. Parsons and Raymond (2014) noted that neurodegenerative diseases, including epilepsy, are characterized by cell death resulting from extrasynaptic NMDA receptor activation following enhanced glutamate spillover, glutamate release from glia, and/or upregulation of NMDARs at extrasynaptic sites. Using N-acetylcysteine (NAC), Deepmala et al. (2015) have demonstrated the potential to treat several psychiatric and neurological disorders, including epilepsy, by attenuating pathophysiological processes relating to these disorders, including neuroinflammation and dysregulation of glutamate and dopamine. Overall, the literature has played an essential and arguably pioneering role in studying glutamate in epilepsy, increasing the volume of subsequent literature in this field.

### Analysis of co-cited references

**FIGURE 7:**
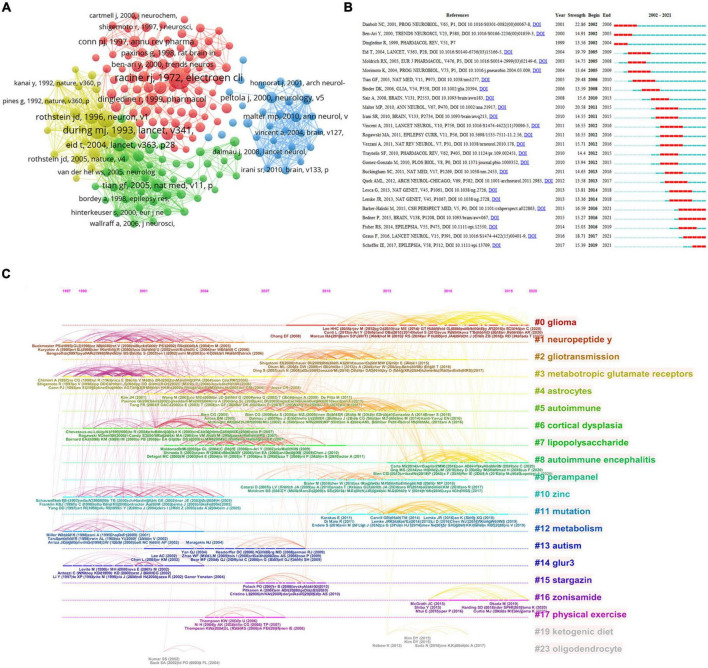
Visualization of co-cited literature analysis. **(A)** Network of co-citations. **(B)** Representative burst citations in the top 25 papers with the most powerful citation burst. **(C)** Timeline distribution of clusters.

The citation network focuses on research topics closely associated with specific fields. Due to the large Nc, the minimum Nc for a reference is 43. Amongst the 154,722 references retrieved, 190 were selected for co-citation analysis (Figure 7A). Citations, represented by the node’s size, denote the total number of co-citations of a document. These papers were divided into different clusters by using different color nodes. Cluster 1 had 85 references (in red), mainly focusing on the role of glutamate and glutamate receptors in epilepsy and the contribution of animal models to the understanding of epileptogenesis. Cluster 2 (in green) concentrates on how glial cell-mediated changes in excitability and inflammation lead to epilepsy. Cluster 3 (in blue) centered on antibodies to voltage-gated potassium channel (VGKC) complexes, NMDARs and AMPARs, and glutamic acid decarboxylase (GAD) associated with limbic encephalitis (LE) and epilepsy. Cluster 4 (in yellow) clarified the critical role of glutamate transporters in epilepsy. Through clustering, most studies were found to focus on glutamatergic mechanisms associated with seizures and epilepsy. Figure 7B shows the top 25 references of the most powerful reference. The studies of Tian et al. (2005) demonstrated the highest burst strength (29.48). They described the relationship between glutamate released by pathologically activated astrocytes and epileptogenesis (Tian et al., 2005). The studies of Danbolt (2001) also possessed higher burst strength (22.86) than those of others. In his paper, he revised the function of glutamate transporters in glutamate uptake and the modulation of neurotransmission, as well as providing glutamate, glutathione, and protein. Furthermore, the research of Malter et al. (2010) showed high burst strength. The authors reported a form of non-paraneoplastic LE defined by high-titer GAD antibodies. In addition, the study of Graus et al. (2016) had an intense citation burst (18.71) in recent years. The authors developed a practical syndrome diagnosis method based on neurological evaluation and routine examination, guiding the differential diagnosis of AE (Graus et al., 2016). The article of Barker-Haliski and White (2015) also showed an intense citation burst (16.59). The authors introduced the relationship between glutamatergic mechanisms and epilepsy (Barker-Haliski and White, 2015). They suggested new targets involving those on microglia and astrocytes, which may provide a means to regulate glutamate, thus bypassing some of the obstacles previously targeted at glutamate receptors themselves (Barker-Haliski and White, 2015). Finally, the works of Scheffer et al. (2017) demonstrated a significant increase in citations. The International League Against Epilepsy updated the terminology and classification of epilepsies (Scheffer et al., 2017). Figure 7C clarifies the most typical references for burstness. The top 10 clusters of co-cited references were “glioma,” “neuropeptide y,” “gliotransmission,” “mGluRs,” “astrocytes,” “autoimmune,” “cortical dysplasia,” “lipopolysaccharide,” “autoimmune encephalitis (AE)” and “perampanel.”

### Keywords analysis

**FIGURE 8:**
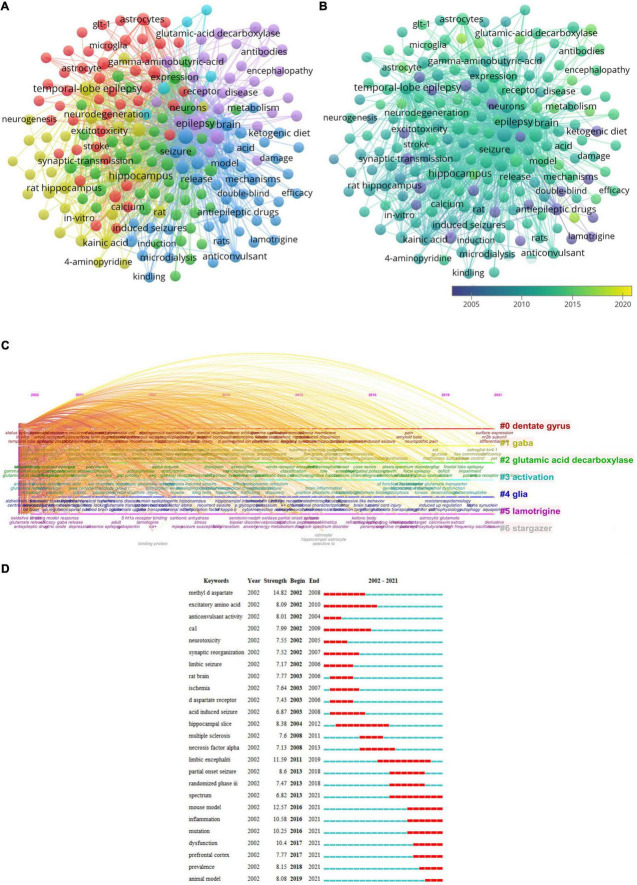
Visualization analysis of keyword. **(A)** Network map of keywords. **(B)** Keyword visualization based on APY. **(C)** Cluster analysis of keywords based on timeline distribution. **(D)** Top 25 representative keywords with the strongest burstness.

In addition to retrieval terms, keywords of 4,891 papers were analyzed (Figure 8). Cluster 1 (46 items, red) was mainly about excitotoxicity induced by glutamate and neuroprotection by glutamate transporters expressed by glial cells. Cluster 2 (46 items, green) primarily reflected how glutamate receptors modulate synaptic transmission. Cluster 3 (44 items, blue) focused on AEDs. Cluster 4 (31 items, yellow) was mainly about animal model studies of epileptogenesis in TLE. Cluster 5 (30 items, purple) primarily reflected autoimmune diseases associated with seizures and epilepsy. Cluster 6 (5 items, cyan) was mainly about *in vivo* studies of epilepsy, such as involving magnetic resonance spectroscopy (MRS) (Figure 8A). The top frequent keywords were “epilepsy,” “glutamate,” “TLE,” “hippocampus,” and “seizures.”

VOSviewer divided all keywords into different colors based on the average publication year (APY, Figure 8B). The main keyword in the latest years was “perampanel” (cluster 3, APY: 2017.19), followed by “neuroinflammation” (cluster 1, APY: 2016.79), “inflammation” (cluster 1, APY: 2015.41), and “LE” (cluster 5, APY: 2015.41). Besides, “dysfunction” (cluster 5, APY: 2015.36), “animal models” (cluster 4, APY: 2015.34), and “mutations” (cluster 5, APY: 2015.33) were the new significant topics in this field. Additionally, “dentate gyrus,” “GABA,” “GAD,” “activation” “glia,” “lamotrigine,” and “stargazer” have been the research focus on glutamate in epilepsy for a long time (Figure 8C). Meanwhile, the terms “spectrum,” “mouse model,” “inflammation,” “mutation,” “dysfunction,” “prefrontal cortex,” “prevalence,” and “animal model” were the hotspots during the last 3 years, as shown in Figure 8D.

Mainline of Figures 8A–D in joint aimed to explore the glutamatergic system associated with seizures and epilepsy. More studies on epilepsy will be performed in the future. In addition, researchers can investigate the mechanism of pathogenesis in epilepsy through basic experiments. So, more means for treating epilepsy will be developed.

## Discussion

As the first bibliometric research on the global study of glutamate in epilepsy, our study performed bibliometric analysis to explore research hotspots and trends of glutamate in epilepsy using the WoSCC database, CiteSpace, and VOSviewer. Four thousand eight hundred ninety-one publications were searched. While the number of published papers fluctuated slightly over the past 20 years, an overall trend toward more published papers was found according to the polynomial fitted curves. This finding suggested that an increasing number of scholars have become interested in the role of glutamate in epilepsy. Our research results show that both research basis and clinical research are involved in this kind of articles, mainly elaborating the role of neurotoxicity and inflammation caused by glutamate in the pathogenesis of epilepsy, which provides a basis for clarifying the pathogenesis and developing drugs and clinical means to treat epilepsy.

The number of publications (Np) and citations without self-citation (Nc), often applied to represent bibliographic materials, were included as bibliometric indicators. In general, as the two significant angles to assess research level, Np is used to measure productivity, and Nc is used to express impact. Recently, H-index has been increasingly used to evaluate researchers’ academic contributions and predict future scientific output (Bertoli-Barsotti and Lando, 2017). By finding the threshold linking Np and Nc, the H-index unifies productivity and influence (Zhao et al., 2016), which could also estimate the publication output of a country or a journal, etc. (Kokol et al., 2021). Besides, the impact factor (IF) is a powerful tool to measure the impact and quality of journals (Villaseñor-Almaraz et al., 2019). The Global Citation Score (GCS) is considered the Nc of a paper on a global scale. It is an essential indicator of an article’s Contribution To The Field of knowledge, and a high GCS indicates a high level of interest from scientists worldwide (Gao et al., 2022).

Publications are distributed worldwide, but the productivity in many areas is not high. Figure 3A shows the geographical distribution of global publications related to glutamate in epilepsy studies. The United States had the greatest Np (*n* = 1,680), followed by China (*n* = 509), Germany (*n* = 468), and Italy (*n* = 375). Amongst the top 10 countries/territories, the United States ranked first in Np, indicating that it is a country very rich in glutamate in epilepsy treatment. The fact that four institutions and one scholar from the United States made the top 10 in glutamate research in epilepsy showed that this country owns the most distinguished affiliations, as well as professional researchers, helping to clarify the reason that the United States had such an impact in the topic over the past 20 years.

Compared with China, the H-index and Nc of the United States are relatively high because the subject has been studied more intensively than in any other country. This finding suggested that Chinese scholars and disciplinary branches should promote their research quality. Similarly, Brazil demonstrated differences in the quantity and quality of publications.

In terms of affiliations, nearly all of the top 10 institutions are from the top eight countries with the most published papers, and about half of them are in the United States, suggesting the good academic ability of the country in this field. Eid T, Aronica, E., and Smolders, I. are the top three scholars who have published the most glutamate research in epilepsy. Therefore, to keep up with the latest development in this field, more attention should be paid to their work, and higher priority should be given. Eid al Fitr (from Yale University; affiliated institutions: Top 9) wrote most of the papers. The team from his institution has long been committed to studying the mechanism of extracellular glutamate elevation in epilepsy. One of his most cited articles described that astrocytic glutamine synthetase (GS) defects might underlie extracellular glutamate accumulation and epilepsy generation in mesial temporal lobe epilepsy (MTLE) (Eid et al., 2004). In the latest study of his team, the authors developed a mouse model for precise and specific deletion of astrocytes GS in small regions of the brain after birth, which is sufficient to cause epilepsy and impair functional connectivity (Farina et al., 2021). This model is expected to be used in rigorous *in vivo* and *in vitro* studies on the GS function of astrocytes at the level of brain regions and single cells (Farina et al., 2021). Figure 4D showed that Univ Genoa, Russian Acad Sci, Harvard Med Sch, Mayo Clin, Polish Acad Sci, Cent South Univ, Yale Sch Med had the highest influence at the moment. Therefore, these institutions can be selected for research cooperation in this field.

Notably, nine of the top 10 journals with most publications had high IF scores, indicating that publishing studies related to glutamate in epilepsy in high-level journals are not difficult. Epilepsia, Epilepsy Research, Brain Research, and the Journal of Neuroscience (J Neuro Sci) made notable contributions. One possible explanation is the IF of these publications. However, the scientific directions and research topics covered by these journals are believed to be more related to the work of scholars. They are more likely to encourage them to submit research reports to these journals. Epilepsia is the leading, authoritative source of innovative clinical and basic scientific research on epilepsy and all aspects of the disease. Epilepsy Research improves the treatment and diagnosis of epilepsy and epilepsy patients with pharmacology, molecular biology, clinical neurology, neuroimaging, and other aspects of clinical methodology and scientific basis. High-quality articles on clinical and basic research in epilepsy have also been published in Epilepsy Research, with a special emphasis on translational research related to epilepsy as a human disease. The journal provides a forum for communicating the most rigorous and cutting-edge research on epilepsy in different disciplines based on molecular biology, biophysics, and other perspectives. Meanwhile, Brain Research is committed to publishing the highest quality and most influential articles in the constantly developing field of neuroscience. It is a broad-format journal accepting manuscripts from the problems of basic neurobiology to translation and clinical neuroscience. It provides contemporary topics in neuroscience, which are of particular innovation and interest, such as neurodegenerative diseases and dementia, psychiatric diseases, autism spectrum disorders, neuromodulation, event-related potential, functional magnetic resonance imaging, and other “windows into brain” stem-cell biology/neurodevelopment. The journal further devotes itself to studying gender differences as an influential cross variable in these fields of interest. In addition, the J Neuro Sci is a multidisciplinary journal with published papers on a wide range of topics of general interest to neuroscientists. Its research topics currently include behavior/cognition, cell/molecule, development/plasticity/repair, neurobiology of diseases, and systems/circuits. As these journals are professional journals with high popularity and influence, scholars may be more likely to publicize their ideals or views in the scientific field to discuss and exchange ideas with their peers to improve their academic level and scientific ability. Finally, the review cycle of these journals is relatively short. Therefore, scholars are more willing to submit articles to them. Based on this trend, the journals shown in Table 3 may still be the “main channel” for future research results in this discipline. It also encourages scholars interested in this topic to read these publications more carefully. Furthermore, most of the research achievements in this field have been published in the journals related to neuroscience. Therefore, if new research achievements in this field need to be published, it is a good choice to contribute to this type of journal, such as Epilepsia, Epilepsy Research, Brain Research, Journal of Neuroscience, Neuroscience, Neuropharmacology, Journal of Neurochemistry, Neurobiology of Disease, and et al.

Articles of three scholars were cited over 380 times (Pascual et al., 2012; Mehta et al., 2013; Zhang et al., 2016). Zhang et al. (2016) had the highest NC, (854). The author developed a method that could acutely purify adult and fetal human astrocytes and obtained transcriptomics profiles of purified human CNS (central nervous system) cell types (Zhang et al., 2016). Given the pivotal role of glia, particularly astrocytes, in glutamatergic mechanisms associated with epilepsy, the transcriptome datasets, and purification methods could be valuable resources for studying human astrocyte biology and exploring new treatments for epilepsy (Zhang et al., 2016). The research results were published in Neuron (IF = 18.688), which were jointly completed by 6 research institutions including Stanford University School of Medicine, Stanford University Medical Center, University of California in the United States and et al. The first cell type specific molecular maps of the brains of healthy and sick patients were shown in their data. Therefore, this study has been cited the most times, indicating that other scholars highly affirmed his work. Mehta et al. (2013) who were from ISF College of Pharmacy in India, summarized the prominent role of glutamate in excitotoxicity in various neurodegenerative disorders, such as epilepsy. Furthermore, they emphasized the downstream triggering events, such as calcium overload, eicosanoic acid pathway, reactive oxygen species (ROS), nitric oxide (NO), chloride homeostasis, and mitochondrial dysfunction, which sustain neuronal excitation. Searching for links between the molecular pathways could enable investigators to test novel therapies. The research results were published in the European Journal of Pharmacy (IF = 5.195). They emphasized that the key way to trigger excitotoxicity was that glutamate depolarizes the membrane of neurons, thus stimulating the accumulation of intracellular calcium and provided a new idea for the treatment of epilepsy.

Pascual et al. (2012) pointed out that the activation of microglia by lipopolysaccharides (LPS) triggers the astrocyte-mediated release of glutamate, which regulates excitatory neurotransmission through mGluRs. The activation of microglia is the main stage of brain inflammation (Pascual et al., 2012). Their work provides a basis for understanding inflammation’s molecular and cellular cascades triggering seizures, which were completed by Institut de Biologie de l’Ecole Normale Supérieure, Institut National de la Santé et de la Recherche Médicale and Centre National de la Recherche Scientifique in France and published in Proceedings of the National Academy of Sciences of the United States of America (PNAS, IF = 12.779). Considering the pathological activation of microglia and the changes in neurotransmission are early symptoms of most brain diseases, their work also provides a basis for understanding synaptic dysfunction in neuronal disorders and important physiopathological relevance among most encephalopathy.

Co-cited references can reflect the degree of connection and structural relationship between references and reveal the thematic similarity of documents from the perspective of citations, as well as the relationship among them. It can be shown from the co-cited references that early detection of the antibodies would be involved in the differential diagnosis of LE and undertake immunotherapy trials (Malter et al., 2010). The practical syndrome diagnosis method based on neurological evaluation and routine examination could eventually prompt immunotherapy (Graus et al., 2016). And future efforts to treat patients with epilepsy with glutamatergic-centric treatments have increased potential (Barker-Haliski and White, 2015). Figures 7C, 8C show hotspots, such as “dentate gyrus,” “glioma,” “GABA,” “AE,” “GAD,” “activation,” “mutation,” “glia,” “lamotrigine,” and “zonisamide.” For many years, the dentate gyrus has been the focal point of research on the molecular, cellular, and network mechanisms that underlie epileptogenesis in TLE (Dudek and Sutula, 2007). Two hypothesized mechanisms have received particular interest and experimental support: (1) selective loss of vulnerable interneurons within the hilus region and (2) the establishment of new recurrent excitatory circuits after sprouting mossy fibers (Kapur et al., 2022; Ramos et al., 2022). Tumor-associated epilepsy and gliomas share pathophysiological mechanisms contributing to ictogenesis and tumor progression (Huberfeld and Vecht, 2016). One major mechanism is excessive glutamate signaling (Dunn-Pirio et al., 2018). Invasion and proliferation are stimulated by high levels of glutamate, causing epileptic discharge and excitotoxicity, thereby promoting the bulk expansion of tumors (Lange et al., 2021). Excitation and inhibition imbalance have been proposed as a mechanism for ictogenesis and epileptogenesis. An imbalance occurs because of extracellular glutamate buildup in the brain and reduced GABA concentration, resulting in excitotoxicity, seizure, and cell death (Sarlo and Holton, 2021). According to Quek et al. (2012), epilepsy is a common symptom of autoimmune nervous system diseases, especially AE. Anti-neuronuclear antibody type 1 autoantibody, collapse protein response mediator protein 5, ma2, antibodies to VGKC complex, GAD65, NMDA, and AMPARs can be identified in AE (Quek et al., 2012). GAD65 catalyzes the synthesis of GABA, the main inhibitory neurotransmitter of the central nervous system (CNS) (Malter et al., 2010). GAD65 antibodies can inhibit the enzymatic action of GAD65, suggesting detection of autoimmune-based drug-refractory epilepsy and the early initiation of immunotherapy may improve epilepsy outcomes (Peltola et al., 2000). Keyword analysis shows that that the research associated with glutamate in epilepsy mainly shows solicitude for glutamatergic system excitotoxicity of epilepsy in the temporal lobe, specifically in the hippocampus. Furthermore, understanding of glutamate transporters could contribute to an enhanced understanding of clinically important conditions and potentially improve treatments (Danbolt, 2001).

A critical article (cited 388 times, Table 5) has demonstrated that the microglia activated by LPS cause astrocyte-mediated glutamate release, which regulates the activity of neurons through neuronal mGluR5 (Pascual et al., 2012). Such a glio transmission mechanism may have important pathological relevance in most brain diseases. Furthermore, Devinsky et al. (2013) and Todd and Hardingham (2020) evaluated the role of glia-induced hyper-excitability and inflammation in epilepsy and revealed that GLT-1 and GLAST (human forms: excitatory amino acid transporters 1 and 2, respectively) expression are downregulated in astrocytes in epilepsy. Therefore, impaired glutamate uptake by astrocytes may increase epileptic hyper-excitability (Devinsky et al., 2013). Interestingly, astrocytic mGluR3 expression, which regulates the expression of GLT-1 and GLAST, is upregulated in TLE (Barker-Haliski and White, 2015). Hence, astrocyte glutamate uptake is enhanced, indicating a compensatory response to prevent seizures. The reduced expression of GS in astrocytes associated with TLE elevates basal glutamate levels and rapid synaptic GABA depletion (Ortinski et al., 2010; Sandhu et al., 2021). Thus, decreased astrocyte GS may have important functions in epileptic susceptibility. Glial-cell-mediated inflammation plays a role in epilepsy and the pathogenesis of epilepsy. Activated astrocytes release interleukin-1B (IL-1B) and High mobility group box 1 proteins, which act through the IL-1 receptor/Toll-like receptor (IL1R/TLR) signaling in glia and neurons (Maldonado et al., 2003). This signaling activates nuclear factor kappa B (NF-κB), thus upregulating the NF-κB-dependent transcription of pro-inflammatory genes (Kamasak et al., 2020). Furthermore, IL1R/TLR signaling activates phosphorylation of the GluN2B subunit of the NMDA receptor and enhances neuronal Ca^2+^ influx, promoting excitability and excitotoxicity (Golub and Reddy, 2022). Activated microglia cooperate with astrocytes to release tumor necrosis factor (TNF)-α and other cytokines, thereby promoting astrocyte glutamate release, which leads to cell loss and seizures (Tan et al., 2021). Inflammatory molecules released by glia, such as TNF-α, can contribute to hyper-excitability by inducing changes in glutamate receptor subunit expression on the neuronal surface, thus causing glutamatergic neurotransmission to increase (Vezzani et al., 2013). Proinflammatory chemokines and cytokines released by astrocytes in epilepsy lead to blood-brain barrier dysfunction, followed by the albumin-mediated downregulation of GLT-1 through the transforming growth factor β pathway and eventually reduced glutamate astrocyte uptake (Diniz et al., 2020). In general, glial cell-mediated excitatory and inflammatory changes lead to epilepsy. Targeting these cytokines and associated signaling molecules is a new option for developing epilepsy therapy.

In less than 65–70% of patients with epilepsy, AEDs effectively control seizures (Pawlik et al., 2021). Novel AEDs, including zonisamide and lamotrigine, have been developed and have a broader spectrum of activity in specific epileptic syndromes and seizure types (Rosenow et al., 2012; Mula, 2013). Broad-spectrum AEDs can act through multiple mechanisms (Schmidt and Schachter, 2014). As a monotherapy and adjunct therapy for epilepsy, lamotrigine is a second-line antiepileptic drug and differs chemically and pharmacologically from other AEDs (Barrera-Bailon et al., 2017). By selectively blocking voltage-sensitive sodium channels, lamotrigine inhibits sodium current and stabilizes neuronal membranes by preventing the release of excitatory neurotransmitters, primarily glutamate (Sills and Rogawski, 2020). Additionally, lamotrigine blocks AMPA glutamate receptors at pharmacologically relevant concentrations (Lee et al., 2008; Rogawski, 2013), although the data of Fukushima et al. (2020) have suggested that lamotrigine inhibits NMDA glutamate receptors at high concentration ranges. Zonisamide blocks voltage-sensitive calcium and sodium channels, upregulates the glutamate transporter (excitatory amino acid carrier-1), inhibits glutamatergic neurotransmission, and downregulates the GABA transporter-1, showing antiepileptic efficacy and tolerance as an adjunct to other AEDs in a double-blind randomized and multinational, phase III study in children aged 6–17 years with partial seizures (Hoy, 2014). Furthermore, the inhibitory effects of zonisamide on gliotransmitter release (such as L-glutamate and ATP) are mediated by preventing astroglial hemichannel activity with connexin43 expression at the plasma membrane (Fukuyama et al., 2020c). In addition, the pathogenesis and pathophysiological basis of nocturnal paroxysmal dystonia in autosomal dominant sleep-related hyperkinetic epilepsy (ADSHE) is the hyperactivation of glutamatergic transmission in the thalamic hyperdirect pathway (Fukuyama et al., 2020a). By activating mGluRs group II in the hyperdirect pathway, zonisamide inhibited glutamatergic transmission (Fukuyama et al., 2020b).

With the development of glutamate research in epilepsy, some new research fields are becoming increasingly interesting. Figure 8A shows the co-occurrence analysis of keywords. The concurrent purpose is to assess the links between recorded items. It is considered a useful tool for predicting evolution and a topic of great interest in specific academic fields. A network diagram of co-occurrence contacts was created by evaluating keywords in all included publications. Finally, six possible research directions were identified as follows (Figure 8A): “mechanism of excitotoxicity induced by glutamate and neuroprotection by glutamate transporters,” “the modulating mechanism of synaptic transmission by glutamate receptors,” “AEDs,” “animal models study of epileptogenesis in TLE,” “autoimmune diseases associated with seizures and epilepsy,” and “*in vivo* studies.” The common keywords with the most occurrences were “epilepsy,” “glutamate,” “TLE,” “hippocampus,” and “seizures.” TLE is one of the most common types of DRE (Engel et al., 2012). The sclerotic hippocampus is key in generating and maintaining temporal lobe seizures (Grote et al., 2022). The hippocampus is an important region for studying the pathological mechanisms of epilepsy. Therefore, researchers have been increasingly focusing on hippocampal studies; thus, relevant studies are expected to increase further. In overlay visual graphics comparable to co-occurrence graphics, items are colored differently by the average appearance time of items. It can immediately track research progress and predict future hot topics. The different colors in Figure 8B represent the appropriate publication year. The findings show that autoimmune diseases associated with seizures and epilepsy accounted for a large portion of blue and green after 2011. It is consistent with the year (2011) when the keyword “LE” in Figure 8D began, indicating that after 2011, more studies concentrated on autoimmune epilepsy associated with glutamate. The keyword “randomized phase III” and “spectrum” in Figure 8D started in 2013, indicating that after 2013, more studies concentrated on the clinical studies of AEDs targeting the glutamatergic system. NMDA and AMPARs antagonists may play a role in antiepileptic drug development. However, NMDA receptor antagonists have failed to demonstrate sufficient efficacy and safety for therapeutic use, and only perampanel, as an AMPAR antagonist, has a new mechanism of action and potential broad-spectrum efficacy (Potschka and Trinka, 2019). Four phase-three randomized controlled trials provide evidence of the efficacy of perampanel (French et al., 2012, 2013, 2015; Krauss et al., 2012). Perampanel is a useful adjunctive therapy for refractory focal seizures with or without secondary generalization and major generalized tonic-clonic seizures (Potschka and Trinka, 2019). Figure 8D shows the keywords with the highest burst intensity in the last 3 years: “spectrum,” “mouse model,” “inflammation,” “dysfunction,” “mutation,” and “prefrontal cortex.” Therefore, experimental models have provided an approach to examine the basic molecular and cellular glutamatergic mechanisms of epileptogenesis in this period. Investigators have paid great attention to inflammation and synaptic dysfunctions, providing a basis for understanding the modulation of glutamatergic mechanisms associated with pathologies in epilepsy. Developing novel medicines with unique action mechanisms for alleviating DRE and targeting glutamatergic systems has become a significant field. Given that the prefrontal cortex is a crucial part of attention and inhibitory control (Simani et al., 2020), it is a vital region for investigating the pathological mechanisms of epilepsy. The alteration of glutamate levels in the prefrontal cortex has been controversial. Using quantitative MRS, Tan et al. (2018) found that in TLE patients, no obvious change in the sum of glutamate and glutamine (Glx) was observed in the dorsolateral prefrontal cortex (DLPFC). According to Tan et al. (2018), spectroscopic abnormalities in the frontal lobe are due to seizure propagation originating in the temporal lobe. Nevertheless, a proton MRS study by Wang W. N. et al. (2021) has suggested that sleep-related hypermotor epilepsy (SHE) is associated with increased Glx concentration in the right DLPFC; their findings may contribute to the understanding of the neurobiochemical mechanisms underlying SHE glutamate concentrations may greatly differ among epilepsy syndromes, and this difference should be considered when making conclusions according to a single study of a single subpopulation of patients with epilepsy. In the past few years, the routine pathway linking epilepsy and inflammation has been gradually identified.

In recent years, more and more attention has been paid to the role of inflammation in epilepsy. Neuroinflammation is the product of epilepsy. Under the pathological conditions characterized by neuritis, the probability of epilepsy is higher. However, the two-way mechanism of the interaction between epilepsy and neuroinflammation remains unclear (Pracucci et al., 2021). Research shows that epilepsy is closely related to inflammation in brain tissue, cerebrospinal fluid and blood of drug resistant epilepsy patients (Pedre et al., 2018). During epilepsy, astrocytes and microglia are activated and TNF-α, IL-6, IL-1β proinflammatory factors were released, and these inflammatory factors constantly activate inflammatory signal pathways such as neurons, glial cells, blood brain barrier, etc., such as NF-KB, COX2, etc., and finally cause extensive biological effects (van Vliet et al., 2018). Inflammatory response and inflammatory factors jointly participate in the destruction of neurotransmitters, neural network reconstruction, touch transmission disorder, blood brain barrier damage, and other processes, which ultimately lead to the continuous increase of neuronal excitability and the continuous decrease of seizure threshold. Therefore, in the future research and clinical treatment, we can take inflammation related molecules and pathways as therapeutic targets to develop new AEDs, and develop effective interventions against inflammatory pathways.

In summary, the hotspots and trends in glutamate in epilepsy studies can be further clarified through visualization of literature and bibliometric analysis. However, our research has limitations. First, SCI expanded data included articles and reviews in English. Second, full texts were not analyzed, and thus some pieces of information were not considered. Finally, our research was delayed because some recently published good articles with low Nc were excluded.

## Conclusion

This research indicated that the Np on glutamate in epilepsy had fluctuated slightly over the past 20 years. However, the general trend is to publish more articles. The study of glutamate in epilepsy offers good research prospects. The United States is a significant producer in this area and has more influence than any other country. The relationship between the glutamatergic system and epilepsy and related clinical studies has stimulated broad public concern. The role of inflammatory pathways and the regulation of neurotransmitters have become potential subjects of great interest in studying glutamate in epilepsy. Enhancing the comprehension of involvement in these mechanisms of pathophysiology in epilepsy will contribute to the optimized use of clinically existing medicines and the identification of novel therapeutic candidates. This study can help scholars further understand the *status quo* of glutamate in epilepsy research from a macro-level perspective.

## Data availability statement

The original contributions presented in this study are included in the article/supplementary material, further inquiries can be directed to the corresponding author.

## Author contributions

WW was in charge of data curation, carried out statistical analysis, and wrote the manuscript’s initial draft. XL and SY carried out supervision. RG, ZR, DY, and KS read and modified the manuscript. All authors reviewed and approved the article’s submission.
